# The effectiveness of counseling based on acceptance and commitment therapy on body image and self-esteem in polycystic ovary syndrome: An RCT

**DOI:** 10.18502/ijrm.v13i4.6887

**Published:** 2020-04-30

**Authors:** Fatemeh Moradi, Akram Ghadiri-Anari, Ali Dehghani, Seyed Reza Vaziri, Behnaz Enjezab

**Affiliations:** ^1^Student Research Committee, Faculty of Nursing and Midwifery, Shahid Sadoughi University of Medical Sciences, Yazd, Iran.; ^2^Department of Internal Medicine, Diabetes Research Center, Shahid Sadoughi University of Medical Sciences, Yazd, Iran.; ^3^Department of Biostatistics and Epidemiology, Public Health School, Shahid Sadoughi University of Medical Sciences, Yazd, Iran.; ^4^Clinical Psychologist, Private Counseling Center of Negaresh, Yazd, Iran.; ^5^Department of Midwifery, Research Center for Nursing and Midwifery Care, Faculty of Nursing and Midwifery, Shahid Sadoughi University of Medical Sciences, Yazd, Iran.

**Keywords:** Acceptance and commitment therapy, Body image, Self-esteem, Polycystic ovary syndrome, Cognitive behavior therapies.

## Abstract

**Background:**

Polycystic ovary syndrome (PCOS) is one of the most common endocrine and metabolic disorders known with irregular menstruation, hirsutism, alopecia, obesity, infertility, and acne. These symptoms cause a negative effect on the satisfaction of body image, self-esteem, and quality of life in such patients. Recent studies emphasize the need to consider the psychological problems in these women and also the need for appropriate interventions.

**Objective:**

The aim of this study was to determine the effectiveness of group counseling based on acceptance and commitment therapy (ACT) on body image and self-esteem in patients with PCOS.

**Materials and Methods:**

In this randomized controlled trial, 52 women with PCOS were randomly allocated to intervention and control groups (n = 26/each) using the table of random numbers. Group counseling based on the ACT was held in eight sessions of 90 min once a week for the intervention group. The demographic questionnaire, Littleton development of the body image concern inventory and Rosenberg self-esteem scale were completed in both groups before, immediately after, and one month after the intervention.

**Results:**

The mean scores of body image concern (p = 0.001) and self-esteem (p ≤ 0.001) in the intervention group after the intervention and follow-up were significantly different from the control group.

**Conclusion:**

Based on the findings of this study, use of cognitive-behavioral therapies in health care centers is recommended as a complementary method.

## 1. Introduction

Polycystic ovary syndrome (PCOS) is one of the most common endocrine disorders with a prevalence rate of 4-25% depending on the definition (1). The prevalence of this syndrome is estimated at 14.6% in Iran and is affects by the prevalence of obesity (2). PCOS is characterized by the heterogeneous presentation of hyperandrogenism, ovulatory dysfunction, and abnormal ovarian morphology (3). Irregular menstruation, hirsutism, alopecia, infertility, and acne are clinical features of PCOS (4) that can effect on feminine identity. Moreover, change in appearance due to these symptoms may lead to behavioral disorders and affect the overall quality of life, sexual satisfaction, and mental health (5). These symptoms also have some negative effects on the body image of the PCOS patients that lead them to feel ashamed of themselves, have low self-esteem (6), and bear substantial pressure (7).

Women more strongly feel dissatisfaction with their appearance as they follow the sociocultural ideal of the female body based on slenderness and thinness (8). These ideals create an intense pressure to conform to beauty standards and consequently have created insecurities via their influence on the perception of self and body image (9). On the other hand, the need for social approval means looking for how someone looks and whether has an attractive body is effective in the self-esteem of young women (8).

Body image means the mental image of the body and each person's attitudes toward the body, appearance, health condition, normal functioning, and sexual desire (10). Negative perceptions of body image in PCOS patients are associated with dissatisfaction of appearance, decreased feminine identity, feeling less sexual attraction, and self-awareness about appearance. The self-esteem of many women is based solely on their body image and thus affects their social activity and interpersonal relationships. Sociocultural factors affect body image and self-esteem, as a result, attitudes toward the body in PCOS women is different. In recent years, research on body image and self-esteem has been developed among Western culture's women but related information from Iranian society is still very scattered (11).

The acceptance and commitment therapy (ACT) is considered as one of the third waves of behavioral therapies and the main purpose of this therapy is to increase psychological ﬂexibility, which includes the ability to live in the moment, pay attention to the main values, and select behavior according to these values while accepting unpleasant experiences (12). Due to the report of the poor body image and low self-esteem in women with PCOS in most descriptive studies and the positive effect of ACT on body image and self-esteem in other conditions (. e.g. infertile women, students with bulimia nervosa, patients with psychosomatic disorders, etc.) (13-21), this study was conducted to determine the effect of group counseling based on ACT on the body image and self-esteem in patient with PCOS.

## 2. Materials and Methods

### Participants

The present study is a randomized controlled trial, parallel and three-stage design (pre-test, post-test, follow-up test). The sample size of the study was 44 (22/group which includes a 20% dropout factor) with the effect size of 0.05, confidence interval of 95% and a power test of 80. So, 52 women with PCOS were randomly assigned into two groups: control (n = 26) and intervention groups (n = 26) using the table of random numbers. The study population consisted of PCOS women in Yazd-Iran.

The inclusion criteria were women aged 18-45 yr, Iranian resident in Yazd, and diagnosed with PCOS according to Rotterdam criteria (22) and endocrinologist diagnosis. The exclusion criteria were women with known psychological problems, such as depression, whole-body laser hair removal, participation in other counseling programs affecting the mind, current pregnancy, women with under 6 months' newborns and major stressors in last 6 months, such as loss of family and divorce. Also, the absence of three sessions or more, laser hair removal during treatment sessions, pregnancy, major stressors and unexpected events in each stage of the plan were dropout criteria. In this study, participation in sport classes was considered as an intervening variable and was considered by asking the units.

### Measurement instruments

#### Demographic information questionnaire

This questionnaire includes items such as age, level of education, occupational status, economic status, marital status, spouse educational level, age, weight, height, number of children, contraceptive method, history of infertility, PCOS symptoms, duration of illness, duration of treatment, medication consumption, and participation in sport classes that were completed at the first meeting.

#### Body image concern inventory

This questionnaire contains 19 items related to dissatisfaction and concern about appearance. In this tool, the subject evaluates her answer to each question on a scale of 1 to 5 (no worries to too much concern). The total score on the scale range from 19 to 95, and higher scores indicate higher dissatisfaction of the body. Littleton and colleagues in 2005 reported 0.93 for Cronbach's alpha coefficient. Also, the validity coefficient of this questionnaire was 0.83 (23). In Iran, the validity and reliability of this questionnaire have been verified (24).

#### Rosenberg self-esteem questionnaire

This questionnaire measures 10 points in a general sense of self- evaluation on a 4-degree Likert range from totally opposite to completely agree. The range of change was 10-40, and higher scores reflected higher levels of self-esteem. Rosenberg reported the reliability coefficient of this questionnaire as 0.82 and the validity of this questionnaire have been verified (25). The validity and reliability of this questionnaire were also reported satisfactory in Iranian society (26).

**Figure 1:**
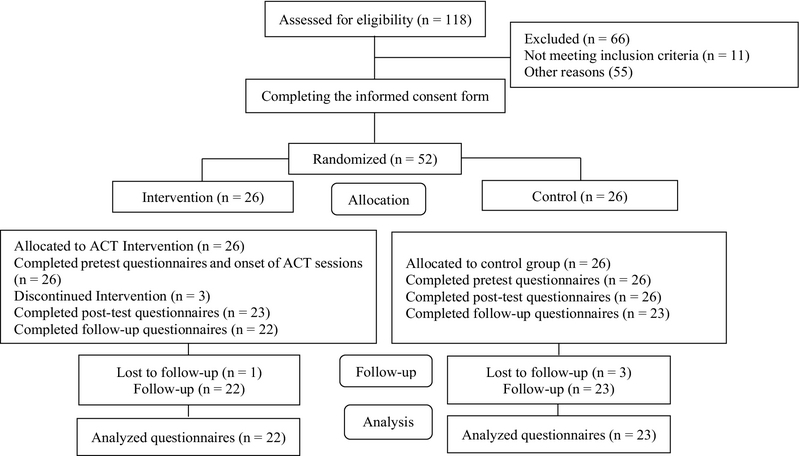
Participant's flow diagram.

The participants of both groups completed the questionnaires before the intervention as a pre-test. Then, sessions based on the ACT were held in 8 sessions of 90 min weekly for the intervention group at the Diabetes Research Center of Yazd city in the summer (August and September) of 2018. Then, they were required to deliver their homework for each session. During this period, the control group received routine treatment including regular physician referrals and taking anti-PCOS medications as before participating in the study. After this, the participants of both groups completed post-test and follow-up the test immediately and one month after the end of the intervention again. Figure 1 shows an overview of participant's flow diagram based on Consort.

The researcher had passed the ACT course and the content of the sessions was checked by a psychologist (Table I).

**Table 1:** The content of sessions based on ACT


**Counseling sessions**	**Goals of sessions**

**Session 1**	Pretest, patients' introduction and review of the aims of sessions
**Session 2**	Teaching the concepts of acceptance and commitment, creating insight into the problem, and challenging the control
**Session 3**	Creating creativity from hopelessness and talk about discomforts and problems that participants have been trying to get rid of
**Session 4**	Creating acceptance and mindfulness by abandoning attempts to control and teaching the diffusion techniques, reviewing the previous session's homework
**Session 5**	Teaching lives based on values and choice, reviewing the previous session's homework
**Session 6**	Assessment of aims, validation of values, purposes, and actions and obstacles to them
**Session 7**	Assessment of values, aims and acts, engagement with eagerness and commitment
**Session 8**	Identification and removal of obstacles to commitment actions, conclusion, post-test (13, 14)

### Ethical consideration

This study was approved by the Ethics Committee of the Yazd Shahid Sadoughi University of Medical Sciences (Code: IR.SSU.REC.1397.004). All participants were informed about the project and completed a written informed consent before participating in the study.

### Statistical analysis

Data were analyzed using the SPSS software (Statistical Package for the Social Sciences, version 16.0, SPSS Inc., Chicago, Illinois, USA). Normality of data was analyzed using the Kolmogorov-Smirnov test. Due to the normal distribution, parametric statistical tests (Independent *t* test and repeated measure test) were used to analyze the data. In addition, categorical variables were compared using the chi-square test. The results of quantitative variables and categorical variables were reported as mean ± standard deviations (SD), and frequency (percentage) respectively. P < 0.05 was considered as significant.

## 3. Results

A total of 52 women participated in the study, who were randomly divided into two groups (intervention group = 26 and control group = 26). However, the number of participants decreased later due to loss of sessions and failure to do homework because of summer trips and long duration of classes (intervention group = 22 and control group = 23) (Figure 1). Data were analyzed and the results showed that the majority of participants had an academic education, irregular menstruation cycles, hair loss, acne, hirsute body and face, no history of infertility, and also were obese and overweight, married, housewives, and did not attend the sport classes (Table II).

The results showed that there was no significant difference in the mean of body image concern scores between the intervention and control groups before intervention, but this difference was significant between the studied groups in two stages of after the intervention and follow-up. In an intra-group comparison of intervention group participants, the results indicated a significant decrease (p = 0.001) in the body image concern scores in all three stages of intervention (Table III).

The mean score of self-esteem before the intervention was not significantly different between the groups. But after the intervention, the mean changes significantly differed between the two groups, and one month after the intervention, there was no significant difference between the two groups. In an intra-group comparison of intervention group, the mean scores of self-esteem in three stages of the intervention was significantly (p ≤ 0.001) changed (Table III).

**Table 2:** Baseline characteristics on subjects in the intervention and control groups


**Variables**		**Control group**	**Intervention group**	P-value*

**Education level**				
	**Diploma and under diploma**	7 (26.9)	10 (38.5)	
	**Academic education**	19 (73.1)	16 (61.5)	<brow>-2</erow> 0.47
**Occupational status**				
	**Employed**	13 (50)	8 (30.8)	
	**Housewife**	13 (50)	18 (69.2)	<brow>-2</erow> 0.15
**Marital status**				
	**Married**	16 (61.5)	21 (80.8)	
	**Single**	10 (38.5)	5 (19.2)	<brow>-2</erow> 0.12
**Spouse's education level**				
	**Diploma and under diploma**	5 (31.2)	9 (42.9)	0.20
	**Academic education**	11 (68.8)	12 (57.1)	
**Infertility history**				
	**Yes**	6 (37.5)	6 (28.6)	0.56
	**No**	10 (62.5)	15 (71.4)	
**Obesity**				
	**Yes**	16 (61.5)	21 (80.8)	0.12
	**No**	10 (38.5)	5 (19.2)	
**Irregular menstruation cycles**				
	**Yes**	21 (80.8)	21 (80.8)	1.0
	**No**	5 (19.2)	5 (19.2)	
**Hirsute face**				
	**Yes**	19 (73.1)	18 (69.2)	0.76
	**No**	7 (26.9)	8 (30.8)	
**Hirsute body**				
	**Yes**	10 (38.5)	11 (42.3)	0.77
	**No**	16 (61.5)	15 (57.7)	
**Acne**				
	**Yes**	9 (34.6)	7 (26.9)	0.54
	**No**	17 (65.4)	19 (73.1)	
**Hair loss**				
	**Yes**	15 (57.7)	16 (61.5)	0.77
	**No**	11 (42.3)	10 (38.5)	
Data presented as frequency (%); *Chi-square test				

**Table 3:** Comparison of mean scores of body image concern and self-esteem and its changes before the intervention, after the intervention, and follow-up in two groups


**Variables**		**Intervention group**	**Control group**	P-value*

**Body image concern**				
	**Before intervention**	40.84 ± 13.50	44.07 ± 9.71	0.32*
	**After intervention**	37.04 ± 12.69	44.46 ± 10.68	0.03*
	**Follow-up**	36.22 ± 12.28	43.60 ± 10.91	0.03*
	P-value**	0.001**	0.105**	
**Self-esteem**				
	**Before intervention**	28.34 ± 5.69	29.11 ± 3.37	0.55*
	**After intervention**	31.82 ± 4.96	29.42 ± 3.36	0.05*
	**Follow-up**	30.59 ± 5.40	30.73 ± 4.30	0.92*
	P-value**	≤ 0.001**	0.006**	
Data presented as Mean ± SD; ${}^{*}$Independent *t* test;${}^{**}$Test of within-subject contrasts of repeated measures				

## 4. Discussion

This trial indicated that group counseling based on ACT reduced body image concern of women with PCOS. Givehki and colleagues studied the effect of ACT on body image flexibility and body awareness in psychosomatic disorders patients. According to the results of this study, ACT had a positive effect on body image flexibility and body awareness of these patients (15). Similarly, the results of the present study and Hill and colleagues (16) suggest that ACT improves the body image score, and this improvement is even consistent with the follow-up period. The studies of Abbasi and colleagues (13), Rasouli Aliabadi and Kalantari (17), and Fogelkvist and colleagues (18) had similar results to this study. However, in these studies, the follow-up of the subjects after the end of the sessions has not been done and the sustainability of the counseling effect has not been evaluated. This improvement of the body image might be explained by the content of this counseling program. In this treatment, using cognitive methods (identifying individual values during sessions, increasing acceptance, and reducing emotional faults) leads to a change in attitude toward the body (13). Another finding was that group counseling based on ACT improves self-esteem in PCOS women. The results of Mortezaie Shemirani and colleagues' study who examined the effect of ACT on the self-esteem of infertile women were in line with the present study in relation to increasing self-esteem after the intervention and follow-up. Similar to the present study, they have reported a reduction in the mean score of self-esteem in the follow-up phase in the intervention group (14). This result might be explained by the fact that participants were obliged to perform regular assignments during the intervention, and therefore the effectiveness of this group counseling on improving body image and self-esteem in the immediate stage after the intervention was more than the follow-up stage. The main difference between this study (14) and the current study was the period of the follow-up phase (three months versus one month). Also, the results of Hinton and Gaynor (19) and Dewhurst and colleagues' (20) studies were in line with the present study and indicated the positive effect of ACT on self-esteem score after the intervention and after one-month follow-up. The results of Saeidmanesh and Babaie's (21) study were in line with the current study with the exception of one-month follow-up phase in the present study. The results of the present study indicate that the mean score of self-esteem was improved in the control group in the post-intervention and follow-up phases that can be related to different factors such as intervening variable in this study (participation in sport classes), reduction in body mass index during the three-month study period, and change in living conditions.

The results of studies of cognitive-behavioral therapies on women with PCOS suggest that these treatments have been effective to improve stress (27), anxiety (27, 28), depression (27-29), quality of life (27), eating problems (28), and decrease in body mass index (29), and treatment by a multi-disciplinary team is effective to improve healthy lifestyle and achieving a long-term weight loss in these women (30). Also, these treatments combined with lifestyle modification compared to lifestyle modification alone have had a greater effect on weight loss and improved symptoms of depression and quality of life in obese or overweight women with PCOS (31). Additionally, group counseling based on the ACT in the present study improved body image concern and self-esteem in women with PCOS in the first follow-up phase, which seems to be ideal. Although in the second follow-up phase, self-esteem decreased in the intervention group participants, this parameter also improved compared to before intervention and was associated with the satisfaction of subjects. According to the participants' ideas in the intervention group, these sessions generally lead to improved mental health and reduced stress and anxiety, which could be associated with weight loss and fertility in the long term.

There were limitations in this study, including the lack of precise control of some factors such as nutritional counseling, which could affect subjects' weight and improvment of body image and self-esteem due to the length of the research period. More precise control of these factors is suggested for the next studies. Due to the decreasing trend in mean self-esteem score, another limitation in the current study was the duration of the follow-up phase. Hence, further studies with long-term follow-up periods is suggested.

## 5. Conclusion

Due to the effectiveness of ACT on psychological health of women with PCOS, its extensive use is recommended as one of the non-pharmacological methods to improve the body image and self-esteem of women with PCOS.

##  Conflict of Interest

The authors declare that there is no conflict of interest to be declared.
