# Corrigendum to “The effectiveness of counseling based on acceptance and commitment therapy on body image and self-esteem in polycystic ovary syndrome: An RCT” [Int J Reprod BioMed 2020; 18: 243–252]

**DOI:** 10.18502/ijrm.v18i12.8143

**Published:** 2020-12-21

**Authors:** Fatemeh Moradi, Akram Ghadiri-Anari, Ali Dehghani, Seyed Reza Vaziri, Behnaz Enjezab

The authors have been informed of an error that occurred on Table III, page 248 in which the correct form of the table is as follows:

**Table III.** Comparison of mean scores of body image concern and self-esteem and its changes before the intervention, after the intervention, and follow-up in two groups

**Table 1:** 


**Variable**		**Intervention group**	**Control group**	P-value*

**Body image concern**				
	**Before intervention**	40.84 ± 13.50	44.07 ± 9.71	0.32*
	**After intervention**	37.04 ± 12.69	44.46 ± 10.68	0.03*
	**Follow-up**	36.22 ± 12.28	43.60 ± 10.91	0.03*
	P-value**	0.001**	0.105**	
**Self-esteem**				
	**Before intervention**	28.34 ± 5.69	29.11 ± 3.37	0.55*
	**After intervention**	31.82 ± 4.96	29.42 ± 3.36	0.05*
	**Follow-up**	30.59 ± 5.40	30.73 ± 4.30	0.92*
	P-value**	< 0.001**	0.006**	
Data presented as Mean ± SD; ${}^{*}$ Independent *t* test; ${}^{**}$ Test of within-subject contrasts of repeated measures				

On behalf of the author, the publisher wishes to apologize for this error. The online version of article has been updated in December 2020.

